# Increased Risk of Migraine in Patients with Chronic Periodontitis: A Population-Based Cohort Study

**DOI:** 10.3390/ijerph18041921

**Published:** 2021-02-17

**Authors:** Yung-Kai Huang, Li-Chiu Yang, Yu-Hsun Wang, Yu-Chao Chang

**Affiliations:** 1 Department of Oral Hygiene, College of Dental Medicine, Kaohsiung Medical University, Kaohsiung 80708, Taiwan; ykhuang@kmu.edu.tw; 2 School of Dentistry, Chung Shan Medical University, Taichung 40201, Taiwan; licioyang@hotmail.com; 3 Department of Medical Research, Chung Shan Medical University Hospital, Taichung 40201, Taiwan; cshe731@csh.org.tw; 4 Department of Dentistry, Chung Shan Medical University Hospital, Taichung 40201, Taiwan

**Keywords:** migraine, chronic periodontitis, population-based, cohort study, Taiwan

## Abstract

Migraine is considered to be a neurovascular disease that manifests as a throbbing headache, possibly caused by the activation of the trigeminovascular system. Several studies have supported the role of inflammation in the pathogenesis of migraine. Chronic periodontitis (CP) is an infectious inflammatory disease triggered by bacterial products evoking an immune response which could result in the destruction of the periodontium. However, little is known about the longitudinal association between CP and migraine. In this study, we designed a nationwide population-based cohort study to investigate the risk of migraine and CP exposure in Taiwan. In total, 68,282 patients with CP were identified from the National Health Insurance Research Database (NHIRD), and 68,282 comparisons were randomly captured and matched by age, sex, monthly income, urbanization and comorbidities. The association between CP exposure and migraine risk was evaluated by Cox proportional hazards regression models. In this study, 785 migraine patients were identified in the CP cohort, and 641 migraine cases were found in the non-CP cohort. The incidence rate of migraine was significantly higher in the CP cohort than the non-CP cohort (adjusted HR: 1.21, 95% CI: 1.09–1.34, *p* < 0.001) during the 13-year follow-up period. Females had a 2.69-fold higher risk for migraine than males (95% CI: 2.38–3.04, *p* < 0.001). In summary, CP is associated with an increased risk of subsequent migraine in Taiwan.

## 1. Introduction

Migraine is a neurological disorder that involves a throbbing headache on one side, usually accompanied by nausea, allodynia and sensitivity to sound or light [1,2]. The prevalence of migraine has been reported to be about 8–15% in the Taiwanese population [3]. Migraine is usually further divided into two subtypes: with or without aura. The definition of aura is recognized as a transient neurological visual, sensory or language symptom that may precede or accompany the headache attack [4]. Migraine has also been sub-classified into episodic migraine and chronic migraine according to the frequency of headache outbreaks [4]. However, the exact mechanisms of the pathogenesis of migraine still remain to be determined. Neuroinflammation via the trigeminovascular pathway is hypothesized to activate several inflammatory mediators triggering the onset of migraine [5]. In addition, the upregulation of these inflammatory factors was found to contribute to the pathogenesis of psoriasis [6], cardiovascular disease [7] and psychiatric disorders [8].

Chronic periodontitis (CP) is an infectious inflammatory disease caused by periodontal pathogens that can escape the host’s immunological defense system, resulting in periodontal tissue destruction, alveolar bone loss and even tooth loss. In Taiwan, the prevalence of periodontitis significantly increased from 1997 to 2013 [9]. CP is not only a local inflammatory disease but also has a connection to influencing systemic conditions. How periodontal disease could contribute to systemic disease has been described via direct and indirect pathways. During the progression of CP, periodontal pathogens could penetrate the epithelial lining of the periodontal pocket and encroach into the circulatory system. Therefore, the circulating pathogens could directly affect the target organs. Apart from this, the inflammatory response from periodontal pathogens or their virulent factors could have indirect systemic effects by an immunological reaction. From the literature review, CP may be linked with vascular systemic inflammation in patients with psoriasis [10], cardiovascular disease [11] and psychiatric disorders [12].

Although CP and migraine share a similar pathogenesis, such as chronic inflammation, endothelial dysfunction and immunological reaction, as described above, the specific association between CP and migraine still needs to be investigated further. Recently, a case–control study matched by age and gender reported the association between CP and chronic migraine in Spain [13]. The results revealed that the prevalence of CP was significantly higher in patients with chronic migraine than those without chronic migraine. The same research team further designed a cross-sectional survey by using a self-reported questionnaire in 12 Spanish headache units [14]. The contents of the questionnaire included socio-demographic, clinical and medical information, comorbidities, daily habits, migraine characteristics and medication. By the logistic regression analysis, the results demonstrated that the prevalence of self-reported periodontitis was significantly higher in chronic migraine patients. However, there was no cohort design and nationwide population to evaluate the association between CP and migraine; it is therefore still necessary to further investigate the causal relationship between the two diseases.

The National Health Insurance (NHI) program in Taiwan is a single-payer insurance system provided by the government. In 2014, almost all of the Taiwanese population was enrolled in this compulsory and universal health insurance program [15]. The National Health Insurance Research Database (NHIRD) contains the de-identified secondary data of comprehensive administrative and medical benefit claim data from the NHI program. With the standard clinical diagnosed criteria by the International Classification of Disease, Revision 9 (ICD-9), the quality of claims of patient charts are randomly cross-checked and strictly surveyed by the peer medical specialists, and this registry-based databank can accurately reflect the health conditions of the general population in Taiwan. NHIRD can provide the real-time condition of Taiwanese population-level data sources for clinical decisions, health care policy-making and epidemiological studies. Therefore, we designed a population-based cohort study to investigate the risk of migraine in patients with CP from NHIRD.

## 2. Materials and Methods

### 2.1. Database

In this study, the Longitudinal Health Insurance Database 2010 (LHID2010), a subset of the NHIRD, was used to clarify the association between CP exposure and migraine risk over a 13-year period. The LHID2010 incorporates 1 million patients’ insurance claims data from the total of 23 million Taiwan NHI beneficiaries in the year 2010. There are no demographic characteristic differences between the LHID2010 and the original NHIRD [16,17]. The LHID2010 contains the most updated claims data of sampled individuals from 1997. The representativeness of LHID2010 has been validated by NHRI [18]. The study protocol was approved by the Institutional Review Board of Chung Shan Medical University Hospital (CS2-15017). Due to the encryption of NHIRD, written informed consent from each of the patients involved was not required. In addition, this research also complied with STROBE (Strengthening the Reporting of Observational Studies in Epidemiology) guidelines for this kind of observational study.

### 2.2. Study Design and Sampled Participants

**Figure 1:**
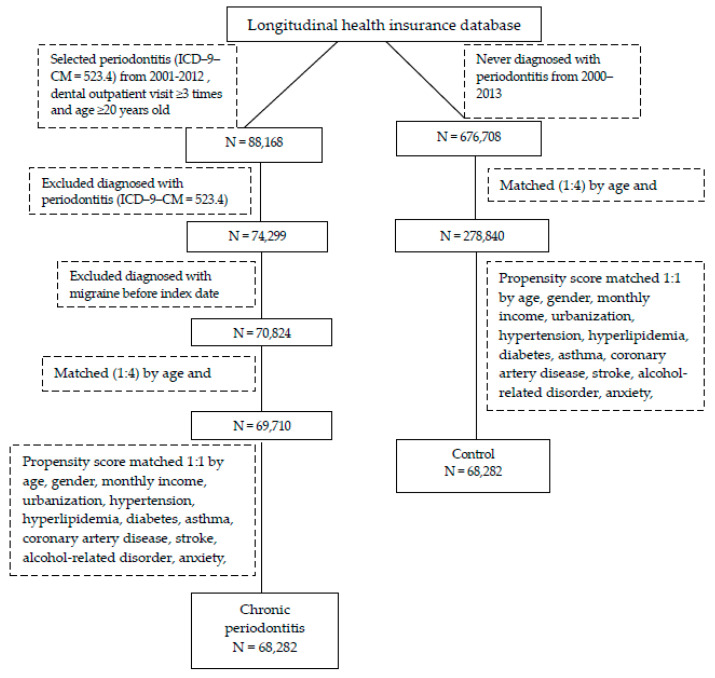
Flowchart illustrating the exclusion and inclusion criteria for this study. ICD-9-CM: International Classification of Disease, Revision 9, Clinical Modification.

The longitudinal association between CP and migraine was interpreted with the cohort design. The study flow diagram of inclusion and exclusion criteria is shown in Figure 1. Briefly, patients with newly diagnosed CP from LHID2010 according to the ICD-9, Clinical Modification code 523.4, from 2001 to 2012 were selected as a CP cohort. The index date was recognized as the first diagnosis date of CP. To increase the validity of CP diagnosis, the sample was limited to patients who had at least three CP diagnoses during dental visits. Moreover, patients diagnosed with periodontitis before 2001 were also excluded.

Subjects without periodontitis were randomly selected from the data set and identified as healthy controls. The comparison group included participations who were not diagnosed with CP from 2000 to 2013. First of all, using 1:4 matching by age, gender was used to provide an index date for the comparison group that corresponded to that of the CP cohort in order to have the same starting time for both groups. The fitting method of 1:4 matching did not eliminate too many subjects with CP and resulted in more control subjects. In order to reduce the confounding bias, we used propensity score matching to select controls. The propensity score of participants, which predicted the probability of CP exposure, was estimated by the logistic regression model. The predictors involved age, sex, monthly income, urbanization and comorbidities at baseline. By matching the propensity score, we could balance the heterogeneity between the two groups. The 1:1 matched comparisons were selected with the same propensity score as the exposure subjects. Patients diagnosed with any type of periodontal diseases before 2001 were also excluded.

### 2.3. Outcome Measurement and Comorbidities

The patients in both CP and non-CP cohorts were followed up until diagnosis with migraine, withdrawal from insurance or the end of 2013; whichever occurred first. Migraine was defined by ICD-9 code 346. Only patients with newly diagnosed migraine were recruited with outpatient visits more than or equal to two times or admission more than or equal to one time assigned by the neurologist.

The potential baseline comorbidities were determined for each patient by ICD-9 codes, including hypertension (401–405), hyperlipidemia (272.0–272.4), diabetes (250), asthma (493), coronary artery disease (410–414), stroke (430–438), alcohol-related disorder (291, 303, 305.0, 571.0–571.3, 790.3, and V11.3), anxiety (300.00), depression (296.2, 296.3, 300.4, and 311), psoriasis (696.1), obesity (278) and insomnia (780.52).

### 2.4. Statistical Analysis

SPSS version 18 (SPSS, IBM, Chicago, IL, USA) was used for all data processing and statistical analyses. To examine the differences between the CP and the non-CP cohorts, a Chi-squared test was used for categorical variables such as sex, age, monthly income, urbanization and comorbidities. The Student’s *t*-test was applied for continuous variables. Hazard ratios (HR) that were used to calculate the effect of CP over time were estimated by Cox proportional hazard models, and the estimates of association were presented with 95% confidence intervals. Probability levels of <0.05 were considered significant.

## 3. Results

The flowchart in Figure 1 illustrates the study design and the selection procedure, while Table 1 shows the demographic characteristics of the study participants. A total of 136,564 participants were enrolled in the study, including 68,282 subjects with CP and 68,282 subjects without CP. The mean ages in the CP and comparison cohorts were 43.7 ± 14.58 and 43.85 ± 14.42 years old, respectively. There was a similar proportion of gender in both cohorts (male vs. female at about 48% vs. 52%). There were also no monthly income and urbanization variations (*p* > 0.05). In addition, the percentage of comorbidities also showed no significant differences between CP and comparison cohorts (*p* > 0.05). No differences between CP and controls were expected due to the propensity score matching.

As shown in Table 2, the number of newly diagnosed migraine patients was 785 in the CP cohort and 641 individuals in the non-CP cohort. CP patients revealed a 1.21-fold increased risk of migraine compared with non-CP patients (HR: 1.21, 95% CI: 1.09–1.34, *p* < 0.001) after adjustment for sex, age, monthly income, urbanization and comorbidities.

The age-specific adjusted HR of migraine decreased 0.52 fold for ages ≥65 years as compared with the 20–64 year-old group (Table 2). The female group had a higher adjusted HR for migraine than the male group (adjusted HR: 2.69; 95% CI: 2.38–3.04, *p* < 0.001). Comorbidities of hyperlipidemia (HR: 1.57, 95% CI: 1.25–1.97, *p* < 0.001), stroke (HR: 2.16, 95% CI: 1.54–3.04, *p* < 0.001), anxiety (HR: 1.75, 95% CI: 1.32–2.33, *p* < 0.001) and insomnia (HR: 2.12, 95% CI: 1.64–2.75, *p* < 0.001) revealed an increased risk of migraine. However, there was no significant risk for migraine with monthly income, urbanization, hypertension, diabetes, asthma, coronary artery disease, alcohol-related disorder, depression, psoriasis and obesity in the adjusted model.

The mean follow-up duration and time to migraine between the CP and non-CP groups is shown in Table 3. The mean follow-up durations of migraine were 8.69 years and 8.6 years for CP and non-CP groups, respectively. The mean times to migraine were 5.05 and 4.87 years for the CP and non-CP groups, respectively.

**Figure 2:**
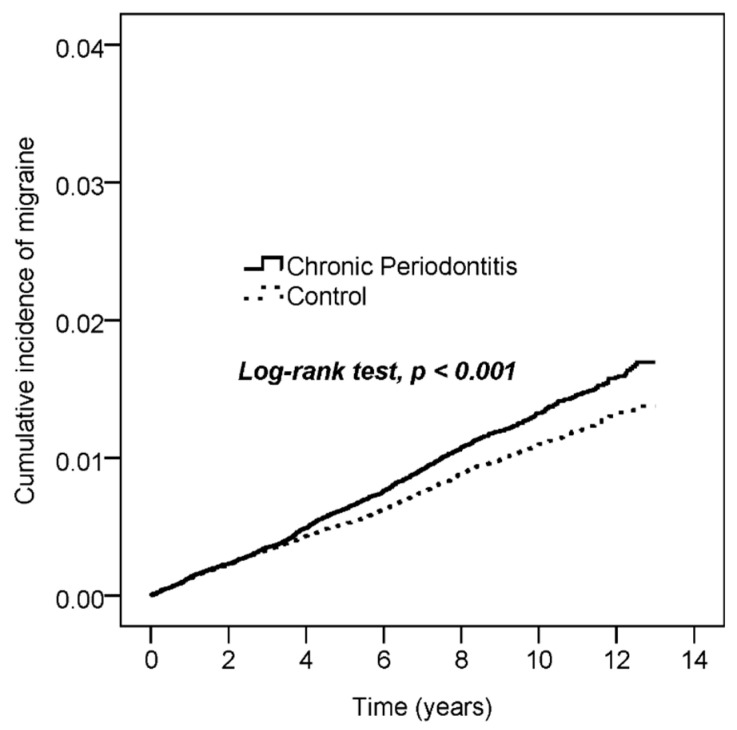
The Kaplan–Meier plot for the cumulative incidence of migraine with chronic periodontitis and control subjects in this cohort study.

Figure 2 shows the cumulative curve of migraine incidence. We used the log-rank test to examine the cumulative incidence of migraine between the groups with and without CP. The results demonstrated that the curve of CP patients was significantly higher than the curve of control subjects (*p* < 0.001).

The stratified analysis of age and gender is presented in Table 4 to compare the risk of developing migraine between patients with CP and the general population. The HR of migraine increased 1.33 fold in CP patients aged 40–64 compared with non-CP patients within the same age group (95% CI: 1.14–1.54, *p* < 0.001). In the male subgroup, the CP patients had a 1.50-fold increased migraine risk (95% CI: 1.21–1.85, *p* < 0.001) compared to non-CP patients. The HR for subsequent migraine was significantly higher in subjects with CP for both sexes compared with that in the control subjects (*p* for interaction = 0.022).

## 4. Discussion

This research is the first large population-based cohort study demonstrating that patients with CP have a significantly higher subsequent risk of migraine than those without CP in a full adjustment model. Kaplan–Meier analysis revealed a significant difference between the CP subjects and controls regarding the risk of developing migraine over the 13-year follow-up period. Two previous studies have reported an association between CP and chronic migraine with a case–control observation study [13] and a cross-sectional questionnaire survey [14]. However, the small samples in the case–control study rendered the results difficult to generalize. In addition, the self-reported questionnaires could not be as valid as the Taiwanese NHIRD. This nationwide registry database can enhance the statistical power to explore the relationship between migraine and CP. In order to achieve high validity, only patients with migraine diagnosed by a neurologist were enrolled. Moreover, we conducted the propensity score-matched control cohort to minimize the selection bias.

Our study analyzed the potential risk factors related to the subsequent occurrence of migraine, such as sex, hyperlipidemia, stroke, anxiety and insomnia. In this study, women displayed a significantly higher risk of migraine than men. The findings were consistent with two review articles that the prevalence of migraine is generally higher among women than among men [19,20]. Migraine is well known to be associated with ischemic stroke [21] and elevated levels of serum lipid such as total cholesterol and triglycerides [22]. Anxiety could induce the onset, or present as a prodrome, of migraine [23]. The relevant literature has shown that disturbances in sleep could contribute to the experience of pain, and the management of sleep disorders could improve the symptoms of migraine [24]. Taken together, these confounding factors were associated with the subsequent occurrence of migraine.

Although the association between CP and migraine still remains unclear, several factors may be involved. The possible mechanisms may involve the same potential inflammatory mediators linked with the two diseases. Leptin, an adipocyte-derived hormone/cytokine, is expressed during inflammation and infection. It could enhance pro-inflammatory cytokine production during infection as an immune-modulator [25]. The concentration of leptin was found to progressively elevate form periodontally healthy individuals to CP, as detected from the gingival crevicular fluid and serum [26]. Moreover, serum leptin levels are increased in patients diagnosed with chronic migraine [27]. Recently, direct evidence has demonstrated that the role of leptin acted as the key biomarker between CP and migraine in a case–control study [28].

Migraine, a painful neurological condition, is believed to be associated with trigeminal sensory system hypersensitivity. Migraine may be the result of neurogenic inflammation [29]. During neurogenic inflammation, neuropeptides and chemical mediators will be released from the peripheral ends of sensory neurons [30]. The vasoactive neuropeptide calcitonin gene-related peptide (CGRP) released during neurogenic inflammation plays an important role in the pathophysiology of migraine [31]. Procalcitonin, a precursor form of the calcium regulatory hormone, is reportedly transcribed from the CGRP [32]. This precursor form is significantly increased throughout the body during conditions of extreme inflammation and is correlated with mortality and the severity of illness, such as sepsis [33]. A cross-section study revealed that serum procalcitonin, the calcium regulatory hormone, was highly elevated in patients with CP and chronic migraine [34]. A previous study demonstrated that patients with migraine have higher periodontal inflammation than a healthy control group [35]. In addition, patients with migraine have more elevated serum CGRP levels in periodontitis than non-periodontitis groups [35].

Migraine is proposed to be linked to vascular inflammation manifested by elevated inflammatory mediators [36]. Increased levels of C-reactive protein or acute-phase reactant pentraxin 3 (PTX3) were found in the systemic circulation of migraine patients [37]. Similarly, systemic elevated levels of PTX3 and soluble fragment of tumor necrosis factor-like weak inducer of apoptosis (sTWEAK) were linked with periodontitis patients [38]. Recently, severe periodontitis was linked with vascular systemic inflammation in patients with chronic migraine via increased levels of acute-phase reactant PTX3 and sTWEAK [39]. Taken together, CP and migraine could be linked with inflammatory processes and vascular endothelial changes via inflammatory mediators of this relationship.

*Helicobacter pylori* (*H. pylori*), the Gram-negative, microaerophilic, rod-shaped bacteria, is considered to be one of the most common bacterial infections associated with chronic gastritis, duodenal ulcer and gastric cancer [40]. In the oral cavity, the microorganisms in dental biofilm and saliva may play a role as a potential reservoir for *H. pylori* [41]. Recently, oral *H. pylori* was found to a potential risk factor for the development of CP [42]. In addition, a systematic review demonstrated a great reduction in *H. pylori* among patients who received periodontal therapy [43]. Interestingly, the cumulative meta-analysis of five studies suggests a trend of more frequent *H. pylori* infection in patients with migraine [44]. Taken together, *H. pylori* could play a role as a key pathogenetic connector between CP and migraine. Regular dental check-ups and prophylaxis might be of benefit for migraine. Dental professions should therefore pay more attention to periodontitis patients with migraine.

The strengths of this study included the nationwide population-based design, a low loss rate in follow-up for both cohorts and the up to 13 years of follow-up, which could reduce the selection bias. However, some limitations need to be addressed for this study. First, the diagnosis of migraine and CP was based on ICD-9 codes. However, there was no corresponding ICD-9 code for any particular type of migraine with and without aura. The severity of CP also could not be obtained from NHIRD. Therefore, the severity of CP as a risk factor for developing a particular type of migraine could not be explored. Second, there is no record of over-the-counter medicines in NHIRD. Those who suffered from migraine without regular anti-migraine agents may have made additional use of painkillers with non-official prescriptions. Third, our report is a registry-based study. There might be some patients with undiagnosed periodontitis in the comparison group. However, the use of propensity score matching in this study may reduce the selection bias and avoid the confounding variates. Fourth, relative confounding factors such as lifestyle, cigarette smoking, alcohol consumption, dietary habits and environmental factors are not provided in the LHID2010. The information about whether pain medications may affect migraine development is also incomplete in this databank. Fifth, the data presented in this study were observational; therefore, potential unknown or unmeasured confounding variables might bias the results. Nevertheless, the single-payer insurance system from the Taiwanese government, the standard clinical diagnosis criteria by ICD-9 and the strictly surveyed peer review of the medical specialists allow the NHIRD to accurately represent the sample for the general population in Taiwan. However, the actual cause–relation or bi-direction speculation is necessary for further investigation. Lastly, the ethnicity of the population in this study was Taiwanese, meaning that the results may not be applicable to other populations.

## 5. Conclusions

The results of this study indicated that individuals with CP exhibited a higher risk of migraine than those without CP. Females had a higher risk of migraine than males. Dental professionals and medical doctors should be more aware of the correlation of CP and migraine. Further clinical and molecular studies will be necessary to confirm our observations and to provide a better understanding of this potential association.

## Figures and Tables

**Table 1:** Demographic data of matched study cohorts.

	Chronic Periodontitis (N = 68,282)		Control (N = 68,282)		
	n	%	n	%	*p*-Value
Age					0.960
20–39	29,409	43.1	29,411	43.1	
40–64	32,425	47.5	32,453	47.5	
≥65	6448	9.4	6418	9.4	
Mean ± SD	43.7 ± 14.58		43.85 ± 14.42		0.051
Gender					0.758
Male	32,884	48.2	32,827	48.1	
Female	35,398	51.8	35,455	51.9	
Monthly income					0.993
<NT$ 20,000	29,242	42.8	29,231	42.8	
NT $20,000–40,000	21,008	30.8	21,028	30.8	
>NT $40,000	18,032	26.4	18,023	26.4	
Urbanization					0.885
Urban	46,450	68.0	46,510	68.1	
Suburban	18,049	26.4	18,027	26.4	
Rural	3783	5.5	3745	5.5	
Hypertension	8101	11.9	8136	11.9	0.770
Hyperlipidemia	3982	5.8	4004	5.9	0.800
Diabetes	3657	5.4	3652	5.3	0.952
Asthma	1036	1.5	1019	1.5	0.706
Coronary artery disease	2510	3.7	2517	3.7	0.920
Stroke	1127	1.7	1133	1.7	0.899
Alcohol-related disorder	137	0.2	124	0.2	0.421
Anxiety	1323	1.9	1303	1.9	0.694
Depression	927	1.4	898	1.3	0.494
Psoriasis	139	0.2	126	0.2	0.424
Obesity	106	0.2	85	0.1	0.128
Insomnia	1461	2.1	1402	2.1	0.265

The Student’s *t*-test and Chi-squared test were used to test the difference of continuous and categorical variables, respectively.

**Table 2:** Risk factor analysis of migraine development.

	No. of Event	Observed Person-Years	ID	Crude HR	95% CI	*p* Value	Adjusted HR ^†^	95% CI	*p* Value
Chronic periodontitis									
No	641	587,048	1.1	1			1		
Yes	785	593,115	1.3	1.21	1.09–1.35	<0.001	1.21	1.09–1.34	<0.001
Age									
20–39	647	522,177	1.2	1			1		
40–64	701	552,912	1.3	1.02	0.92–1.14	0.661	0.98	0.88–1.1	0.776
≥65	78	105,074	0.7	0.60	0.47–0.76	<0.001	0.52	0.4–0.67	<0.001
Gender									
Male	364	567,014	0.6	1			1		
Female	1062	613,149	1.7	2.70	2.4–3.04	<0.001	2.69	2.38–3.04	<0.001
Monthly income									
<TWD 20,000	603	507,213	1.2	1			1		
TWD 20,000–40,000	480	359,766	1.3	1.12	1–1.27	0.058	1.12	0.99–1.26	0.073
>TWD 40,000	343	313,184	1.1	0.92	0.81–1.05	0.226	1.08	0.94–1.24	0.279
Urbanization									
Urban	943	802,066	1.2	1			1		
Suburban	398	313,350	1.3	1.08	0.96–1.21	0.197	1.10	0.97–1.23	0.128
Rural	85	64,747	1.3	1.12	0.89–1.39	0.330	1.13	0.9–1.41	0.290
Hypertension	151	129,261	1.2	0.97	0.82–1.14	0.683	0.91	0.74–1.11	0.358
Hyperlipidemia	101	60,017	1.7	1.43	1.17–1.75	0.001	1.57	1.25–1.97	<0.001
Diabetes	64	57,293	1.1	0.92	0.72–1.19	0.535	0.84	0.64–1.1	0.213
Asthma	26	17,449	1.5	1.24	0.84–1.82	0.281	1.19	0.8–1.76	0.385
Coronary artery disease	54	41,251	1.3	1.09	0.83–1.43	0.542	1.16	0.86–1.55	0.331
Stroke	38	17,652	2.2	1.81	1.31–2.5	<0.001	2.16	1.54–3.04	<0.001
Alcohol-related disorder	4	1993	2.0	1.67	0.63–4.45	0.306	2.13	0.79–5.7	0.133
Anxiety	56	20,483	2.7	2.32	1.78–3.03	<0.001	1.75	1.32–2.33	<0.001
Depression	35	14,555	2.4	2.02	1.44–2.83	<0.001	1.41	1–1.99	0.053
Psoriasis	5	2190	2.3	1.89	0.79–4.55	0.154	2.11	0.88–5.08	0.096
Obesity	1	1540	0.6	0.54	0.08–3.82	0.535	0.46	0.06–3.27	0.437
Insomnia	66	22,490	2.9	2.51	1.96–3.21	<0.001	2.12	1.64–2.75	<0.001

ID: Incidence density, per 1000 person-years. ^†^ Adjusted for age, gender, monthly income, urbanization, hypertension, hyperlipidemia, diabetes, asthma, coronary artery disease, stroke, alcohol-related disorder, anxiety, depression, psoriasis, obesity and insomnia.

**Table 3:** Track time of chronic periodontitis and control cohort.

	Chronic Periodontitis (N = 68,282)	Control (N = 68,282)	*p*-Value
Follow-up duration (years)	8.69 ± 3.08	8.6 ± 3.1	<0.001
Time to migraine (years), N = 1426	5.05 ± 3.15	4.87 ± 3.23	0.280

The Student’s *t*-test was used to test the difference of continuous variables.

**Table 4:** Subgroup analysis of hazard ratios of chronic periodontitis.

Heading	Chronic Periodontitis		Control		HR	95% CI	*p* Value
	N	No. of Event	N	No. of Event			
Age							
20–39	29,409	340	29,411	307	1.10	0.94–1.28	0.234
40–64	32,425	401	32,453	300	1.33	1.144–1.54	<0.001
≥65	6448	44	6418	34	1.24	0.79–1.94	0.342
Gender							
Male	32,884	219	32,827	145	1.50	1.21–1.85	<0.001
Female	35,398	566	35,455	496	1.13	0.999–1.27	0.051

*p* for interaction = 0.022.

## Data Availability

Restrictions apply to the availability of these data. Data was obtained from National Health Insurance database and are available from the authors with the permission of National Health Insurance Administration of Taiwan.
